# Declining pain medicine fellowship applications from 2019 to 2024: A concerning trend among anesthesia residents and a growing gender disparity

**DOI:** 10.1111/papr.13441

**Published:** 2024-11-16

**Authors:** Scott G. Pritzlaff, Naileshni Singh, Chinar Sanghvi, Michael J. Jung, Paul K. Cheng, David Copenhaver

**Affiliations:** ^1^ Division of Pain Medicine, Department of Anesthesiology and Pain Medicine University of California‐Davis Sacramento California USA

**Keywords:** chronic pain management, fellowship training, healthcare demands, pain fellowship applicants, pain medicine, workforce trends

## Abstract

**Introduction:**

The fields of anesthesiology and pain medicine are experiencing significant changes driven by market forces and professional preferences. While demand for anesthesiologists is rising, pain medicine is facing a decline in fellowship applications.

**Methods:**

This study analyzed data from the Electronic Residency Application Service (ERAS) and the National Resident Matching Program (NRMP) from 2019 to 2023, focusing on trends in fellowship applications to pain medicine programs. Additionally, preliminary data from the 2024 match cycle were examined.

**Results:**

There has been a notable decrease in anesthesiology residents applying to pain medicine fellowships, with applications dropping from 351 in 2019 to 193 in 2023. The overall decline in anesthesia‐based applicants to pain medicine fellowships was 45%, signaling the highest detriment among anesthesiology applicants compared to other specialties. Gender disparities have been prevalent, with the absolute number of female applicants decreasing every year since 2019. Additionally, the 2023 match saw a significant number of unfilled programs, with 35 out of 115 programs failing to fill all positions. Preliminary data from the 2024 match cycle suggest this downward trend is continuing.

**Conclusion:**

The decline in pain medicine fellowship applications, particularly among anesthesiology residents, signals potential future workforce shortages and challenges in patient care. Recruitment strategies should include early exposure to pain medicine during residency, enhanced mentorship programs, and robust recruitment efforts (including virtual options). Addressing these issues is essential to ensure enough trained specialists to meet the growing need for pain specialists nationally.

## INTRODUCTION

The fields of anesthesiology and pain medicine stand at a pivotal juncture in 2024, influenced by various market factors shaping their landscape. This article delves into the intricate interplay of these factors, providing a comprehensive analysis of the current trends and dynamics defining the practice of anesthesiology and efforts to train tomorrow's anesthesiologists and pain medicine specialists.

In recent years, the demand for anesthesiologists has witnessed a notable surge, propelled by fluctuating demographics, advancements in medical procedures, the burgeoning healthcare needs of an aging population, and the post‐COVID shifts in medicine.^1^ This surge in demand has not only underscored the critical role of anesthesiologists in ensuring patient safety and comfort during surgical interventions but has also sparked a wave of opportunities within the field. One such opportunity lies in compensation, where anesthesiologists are experiencing increased pay scales offered by private groups and hospital systems.^2^ This trend reflects the recognition of and resulting market demand for anesthesiologists' specialized skills and expertise in the operating room and other medical settings, driving institutions to compete for top talent through competitive remuneration packages. US News and World Report names anesthesiology as the best‐paying job in healthcare (tied with Obstetrics and Gynecology and Oral Maxillofacial Surgery).^3^ In addition, the US Bureau of Labor Statistics places job growth for anesthesia at 2.6%, with 1000 projected job openings between 2023 and 2032.^4^

Furthermore, the landscape of anesthesiology practice is witnessing a significant shift towards flexibility, as evidenced by the emergence of flexible locum tenens offerings or part‐time employment. These arrangements afford anesthesiologists the autonomy to negotiate compensation, lifestyle‐focused scheduling, and flexible work locations, thus catering to their individual preferences, enhancing work–life balance, and decreasing burnout.^5^, ^6^

In contrast, pain medicine has seen an overall decrease in the number of applicants to Accreditation Council for Graduate Medical Education (ACGME)‐accredited fellowship programs between 2019 and 2023.^7^ This trend may result in downstream effects such as workforce shortages, causing longer wait times for pain patients and delays in their care. Current specialists could face further increased workloads, heightening the risk of burnout and early retirement and further exacerbating the shortage. Additionally, reduced interest in pain medicine fellowships could lead to program closures, diminishing the number of new specialists and creating a cycle of decline in training opportunities. Furthermore, a decline in fellowship applicants could stifle research and innovation in pain medicine, limiting advancements in treatments and technologies. Patient care, in the midst of a national opioid crisis and the public health problem of pain, can be adversely impacted. Addressing these issues will require initiatives to enhance the appeal of pain medicine, such as financial incentives, improved work–life balance, and increased awareness of the specialty's importance and rewards.

Amidst positive perioperative anesthesiology trends in salary where trainees are choosing to enter the workforce instead, a concerning pattern has emerged regarding subspecialty preferences within anesthesiology. Specifically, there has been a notable decrease in the number of anesthesiology residents applying to subspecialty programs such as pain medicine. At the same time, there has been a growth of applicants from alternative non‐anesthesia field specialties like physical medicine and rehabilitation (PM&R) and emergency medicine (EM).

## METHODS

Data for this study were sourced from publicly available reports on the National Resident Matching Program (NRMP) and Electronic Residency Application Service (ERAS) websites. Complete reports for fellowship application years 2019–2023 were analyzed. A limited preliminary report of 2024 applicants to ERAS was also evaluated. ERAS and NRMP deidentified all data used in this analysis. This study was exempt by the University of California‐Davis Institutional Review Board (IRB).

Additionally, the authors acquired a specialized report from the American Association of Medical Colleagues (AAMC), offering a comprehensive analysis of applicant specialty backgrounds from 2019 to 2023. This report specifically scrutinized the number of applicants from various medical specialties seeking admission to pain medicine fellowship programs with some demographic information.

The analysis focused on discerning trends in fellowship applications, particularly among anesthesiology residents, and evaluating the broader landscape of pain medicine fellowship programs. This involved reviewing available data on the number of applicants, specialty backgrounds, race, and gender trends for the above cycle. Furthermore, preliminary data from the ongoing 2024 match cycle were gathered and analyzed to ascertain the continuation of observed trends.

## RESULTS

### ERAS applicant trends

As evidenced by the ERAS applications, the pain fellowship landscape has witnessed unforeseen challenges. Several pain fellowship programs remained unfilled in 2023, leading to a “wake‐up” call among program directors.^8^ This tumultuous scenario followed a preceding year characterized by record‐breaking applicant numbers, with 548 applicants vying for available slots, significantly exceeding the available positions in 2022. However, in the subsequent year of 2023, the number of applicants plummeted to 446, signaling a notable decline.^9^

A point of intrigue emerged regarding the role of anesthesia resident applicants in driving this shift. The authors obtained a special AAMC report examining applicant specialty backgrounds from 2019 to 2023 (Table 1). The number of anesthesiology residents applying to pain fellowship has decreased for at least five consecutive years (eg, 2019–2023). Notably, the data revealed a decline in the anesthesia applicant pool from 2022 to 2023, amounting to a 45% decrease in anesthesiology trainees applying to pain medicine fellowships (351 in 2019 to 193 in 2023). This decline is particularly noteworthy considering that even starting in 2022, the pool had exhibited a slight downturn compared to the preceding years of 2019–2021. While the overall decline among all applicants was approximately 14.2% (529 in 2019 and 446 in 2023), the decrease in anesthesiology applicants outpaced this overarching pain applicant pool downtrend.

**TABLE 1:** Applicant trends by specialty in pain medicine 2019–2023 (Source: AAMC).

Primary specialty	Match year				
	2019	2020	2021	2022	2023
Anesthesiology*	351	303	302	293	193
Physical medicine and rehabilitation*	101	124	131	141	134
Neurology*	22	13	11	19	17
Emergency medicine*	10	19	20	26	29
Family medicine*	7	5	11	8	10
Psychiatry*	4	6	8	5	6
Radiology‐diagnostic*	1	2	2	1	0
Internal medicine	6	7	8	3	5
Child neurology	0	1	0	1	1
Osteopathic neuromusculoskeletal medicine	0	0	0	1	1
Otolaryngology	1	0	0	0	0
Public health and general preventive medicine	0	1	0	1	0
Occupational and environmental medicine	0	0	1	0	0
Surgery‐general	0	0	4	2	1
Urology	0	1	0	0	0
Sleep medicine	0	0	1	1	0
Internal medicine/emergency medicine	0	0	1	0	0
Internal medicine/psychiatry	0	1	0	0	0
Pediatrics/anesthesiology	0	1	0	0	0
Internal medicine/anesthesiology	1	0	1	0	1
Transitional year	0	1	4	6	5
Unknown specialty	16	29	35	40	43
Total applicants	520	514	540	548	446

* Denotes board‐eligible specialties as defined by the American Board of Medical Specialities.^10^

Interestingly, amidst the fluctuations observed in anesthesia applicants, the data highlighted the stability of PM&R applications, which showed minimal variation from 2019 to 2023. Non‐traditional applicants, such as EM trainees, have increased nearly threefold between 2019 and 2023. Family medicine, internal medicine, and neurology had too few applicants to establish clear trends (Table 1). However, the ERAS fellowship applicant numbers do not detail the number of applicants who match and ultimately enroll in pain medicine fellowship programs.

Another critical aspect of the pain medicine fellow applicant pool is that racial and gender disparities are emerging amidst the overall decrease in applicants. Pain medicine has not been a field that regularly attracts minorities or other underrepresented groups, and the small numbers of these individuals participating in ERAS and the NRMP do not allow meaningful trends to be deciphered (Table 2). However, most applicants self‐identify as “White” (43% in 2019 and 40% in 2023), with those who identify as “Asian” increasing slightly from 2019 (31%) to 2023 (35%).

**TABLE 2:** The self‐identified race and ethnicity and gender (men/women) breakdown per year for ERAS applications. (Source AAMC).

Match year	2019	2020	2021	2022	2023
Race/ethnicity (N)					
American Indian or Alaska Native	2	1	4	2	2
Asian	159	151	156	168	157
Black or African American	30	23	35	32	32
Hispanic, Latino, or of Spanish Origin	34	38	35	34	37
Native Hawaiian or other Pacific Islander	0	1	1	4	0
White	219	237	243	248	177
Other race/ethnicity	20	21	30	24	27
Unknown race/ethnicity	48	34	42	37	12
Gender (N)					
Men	389	413	418	430	351
Women	131	101	121	118	95
% Female	25.1	19.6	22.4	21.5	21.3

Unfortunately, the gender disparities between self‐identified men and women are worsening with the decrease in overall applicants (Table 2). The number of female applicants has decreased yearly since 2019, with 131 in 2019 and 95 in 2023 (an overall 27.5% decrease). This is disproportionate to the 9.8% decrease in men from 2019 to 2023, with notable fluctuations (increases) in applicant numbers for 2020, 2021, and 2022. The lack of females choosing pain medicine for subspecialty training is a well‐known issue in the field, and current trends further worsen this disparity.^11^, ^12^

### The 2023 match

**FIGURE 1:**
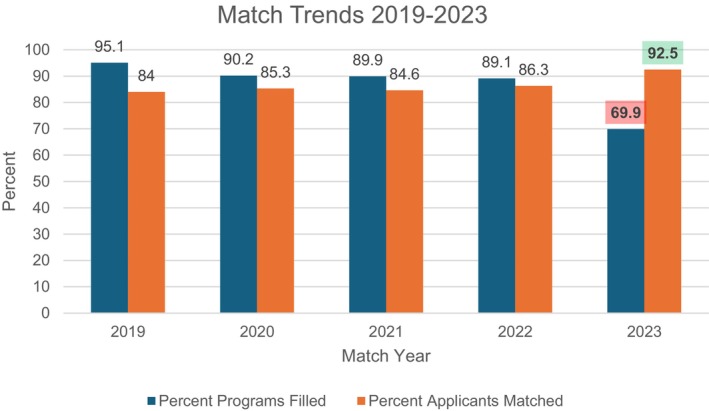
Pain medicine applicant and program match trends, 2019–2023. The 2023 match showed an unprecedented percentage of unfilled programs (Source: NRMP).^13^

The match for pain medicine in the Fall of 2023 proved to be a significant turning point in anesthesia fellow recruitment, drawing the attention of program directors and academic faculty for several reasons. Firstly, after a five‐year trend where applicants typically outnumbered available positions, 2023 was the first time in half a decade that the number of positions offered in the match exceeded the number of applicants. Therefore, the 2023 match witnessed unprecedented unfilled programs (Figure 1). Out of 115 programs, a record 35 went without an entire class of fellows, with 61 unfilled slots (a threefold increase in vacancies compared to 2022).^13^ This surge in unfilled programs compared to previous years (12 in 2022 and 11 in 2021) highlights program directors' challenges in attracting suitable candidates. It also underscores a growing gap between the supply of fellowship positions and the demand from applicants to pursue fellowship training. The downstream effects could result in pain provider shortages and increased responsibilities for matched fellows, such as call burden in the setting of an unfilled fellowship class cohort group.

### 2024 Application cycle

As of May 2024, a review of the preliminary AAMC applicant data for pain medicine shows no notable slowing of this decline. As of April 12, 2024, 410 applicants have applied for fellowship for this cycle. This represents a further 8.1% decline in total applicants from 2023 and an overall 21.2% decline compared to 2019.^9^ Since the majority of candidates submit applications from November to February, it is unlikely that the final number will change dramatically. Race, gender, and specialty data are not reported in these preliminary data. Given this continued concerning trend, the high number of unfilled programs is likely to persist.  

## DISCUSSION

The findings presented in this analysis shed light on significant shifts within anesthesiology, particularly regarding fellowship training and residency programs. The decline in applicants to subspecialty programs, such as pain medicine, is a notable trend observed in recent years. This decline contrasts with the surge in overall demand for anesthesiologists, indicating a shift in professional preferences within the specialty but may be consistent with declining trends in other anesthesiology subspecialties.^14^, ^15^

The emergence of lucrative opportunities within general anesthesiology practice, including competitive compensation and flexible work arrangements, could deter trainees from pursuing subspecialty training. Based on the most recent 2022 provider compensation data from the Medical Group Management Association (MGMA) DataDive program, the median total compensation for pain physicians with a primary background in anesthesiology was $472,333, while the median compensation for anesthesiologists was $498,954. Interestingly, when evaluating non‐anesthesiologist pain physicians, which includes physiatry‐based pain physicians, the total reported median compensation in 2022 was $507,508, while the median compensation for general physiatrists was $325,429.^16^ The prospective allure of a financially lucrative career, especially for anesthesiologists, may overshadow the perceived benefits of fellowship training in pain medicine. Pre‐COVID pandemic, pain medicine was thought to be on the rise with advances in research, robust growth in technology with numerous interventional offerings, including minimally invasive surgical options, growing numbers of pain societies supporting the field, and attractive career options.^17^ In a few short years, this has significantly shifted.

The gender disparities in medicine are widening with the decrease in pain applicants. A study by Doshi et al. investigated the gender distribution within Pain Medicine fellowship programs in the United States from 2017 to 2018, revealing that around 25% of fellows were women. It was found that programs led by female fellowship program directors were 2.40 times more likely to have female trainees.^12^ Furthermore, the study indicated that the presence of female leadership, whether as program directors or division chiefs, correlated with a higher proportion of female faculty, emphasizing the significance of female role models in addressing gender underrepresentation in academic pain medicine. Pain medicine is positioned in the lowest quartile among medical specialties regarding female representation (18% female physicians), just marginally surpassing traditionally male‐dominated fields such as orthopedic surgery (14%) and neurosurgery (17%). In contrast, several other surgical disciplines exhibit higher levels of female participation, such as colorectal surgery (42%), general surgery (37%), and urology (26%). With regard to anesthesiology sub‐specialties, ACGME data reveal that women enroll in all other ACGME‐accredited anesthesiology fellowships at greater rates compared to pain medicine (22%): cardiothoracic anesthesiology (30%), critical care (27%), regional anesthesiology (40%), obstetric anesthesiology (59%), and pediatric anesthesiology (53%). This underscores the importance of thoroughly examining the root causes of this gender gap and initiating proactive measures, starting with the recruitment of diverse trainees.

Lastly, this application shift indicates a potential change in the composition of pain physicians overall, with more opportunities opening for aspiring non‐anesthesiologists to complete pain medicine fellowships. The representation of each class will likely be affected by the decline in the number of anesthesiology trainees applying. Future fellowship classes will likely consist of largely non‐anesthesiology‐trained physician pools. This can signal an opportunity to diversify the composition of fellowship classes nationwide as PM&R, EM, neurology, and others bring a unique perspective to pain's complex and multifaceted nature.

With the discussion of the almost 5‐year decline in applicants to pain medicine fellowships, the question is how to remedy this situation. Programs should offer an early exposure to acute and chronic pain rotations within the first or second year of residency to identify interest given that the fellowship match occurs early in the last year of residency training (and thus, most residents must decide regarding their subspecialty application in their third year of residency). In addition, robust pipeline and mentorship programs must be developed to encourage individuals to consider pain medicine a potential career option, especially for female and minority applicants. Many pain societies offer mentorship programs, including the North American Neuromodulation Society (NANS), the American Society of Regional Anesthesia and Pain Medicine (ASRA), and the American Society of Pain and Neuroscience (ASPN), which can help trainees envision a lifestyle dedicated to pain management. Active participation and representation of pain medicine attendings are crucial for the success of these mentorship programs.

Additionally, while virtual interviews are convenient and save travel time and money for the candidate, they may need to be more engaging for trainees. For example, some programs have utilized a virtual multiple mini‐interview (MMI) format since very few programs have returned to in‐person interviews since the COVID‐19 pandemic.^18^, ^19^, ^20^ Many programs also offer multiple virtual “meet and greet” sessions with current fellows and faculty throughout the recruitment cycle to maintain engagement and give candidates a better understanding of the educational offerings. With the rising popularity of social media, pain programs now maintain a presence on platforms like Facebook, Instagram, 𝕏 (formerly Twitter), and LinkedIn. Maintaining participation in the match is crucial to ensuring a fair and transparent selection process. Per the Association of Pain Program Directors (APPD), offering positions outside the match is not recommended, as this can lead to confusion and create unnecessary competition, disrupting the fairness and clarity of the process. While these are possible solutions to our field's upcoming shortfall, each program must reflect individually and temper expectations regarding the upcoming match. Perhaps the more significant issue is that there will not be enough pain medicine specialists to serve our current and future population. Moreover, the trends in applicant data portend a palpable reduction in the number of sophisticated, specialized clinicians addressing tomorrow's complex pain care needs, placing our patients and communities in a precarious position.

## CONCLUSION

The landscape of anesthesiology practice is evolving, influenced by various market factors and professional preferences. While the overall demand for general anesthesiologists remains high, there needs to be more applicants to subspecialty fellowship programs, notably pain medicine. Understanding the drivers behind these trends is essential for stakeholders within the field to adapt educational and workforce strategies accordingly.

Addressing the challenges of declining fellowship applicants requires collaborative efforts from program directors and medical educators. By fostering a deeper understanding of career preferences and market dynamics, the field of pain medicine can navigate these changes and ensure a robust workforce equipped to meet society's evolving healthcare needs.

## AUTHOR CONTRIBUTIONS

SP, NS, MJ, and CS were involved with the manuscript conception and initial data review. SP, NS, and CS created the tables and figures for the publication. SP, NS, CS, MJ, PC, and DC contributed to drafting, editing, and final approval of the manuscript.

## FUNDING INFORMATION

Funding from the UC Davis Division of Pain Medicine was provided to obtain a special report from the Association of American Medical Colleges (AAMC) outlining specialty‐specific application trends.

## CONFLICT OF INTEREST STATEMENT

Scott G. Pritzlaff MD‐ Consultant: Bioness, SPR Therapeutics, Nalu Medical; Royalties: Oxford University Press, Wolters Kluwer; Education Grants: Medtronic, Nevro Corp, Abbott, Biotronik; the remaining authors have no conflicts to disclose.

## Data Availability

Data available on request from the authors.
